# miR-483-5p orchestrates the initiation of protein synthesis by facilitating the decrease in phosphorylated Ser209eIF4E and 4E-BP1 levels

**DOI:** 10.1038/s41598-024-54154-1

**Published:** 2024-02-20

**Authors:** Siranjeevi Nagaraj, Anna Stankiewicz-Drogon, Edward Darzynkiewicz, Urszula Wojda, Renata Grzela

**Affiliations:** 1 Interdisciplinary Laboratory of Molecular Biology and Biophysics, Centre of New Technologies, University of Warsaw, 02-097 Warsaw, Poland; 2 Division of Biophysics, Institute of Experimental Physics, Faculty of Physics, University of Warsaw, Pasteura 5, 02-093 Warsaw, Poland; 3 Laboratory of Preclinical Testing of Higher Standard, Nencki Institute of Experimental Biology of Polish Academy of Sciences, Pasteur 3, 02-093 Warsaw, Poland

**Keywords:** Cancer, Molecular biology

## Abstract

Eukaryotic initiation factor 4E (eIF4E) is a pivotal protein involved in the regulatory mechanism for global protein synthesis in both physiological and pathological conditions. MicroRNAs (miRNAs) play a significant role in regulating gene expression by targeting mRNA. However, the ability of miRNAs to regulate eIF4E and its phosphorylation remains relatively unknown. In this study, we predicted and experimentally verified targets for miR-483-5p, including eukaryotic translation initiation factor eIF4E and its binding proteins, 4E-BPs, that regulate protein synthesis. Using the Web of Science database, we identified 28 experimentally verified miR-483-5p targets, and by the TargetScan database, we found 1818 predicted mRNA targets, including *EIF4E*, *EIF4EBP1*, and *EIF4EBP2*. We verified that miR-483-5p significantly reduced *ERK1* and *MKNK1* mRNA levels in HEK293 cells. Furthermore, we discovered that miR-483-5p suppressed *EIF4EBP1* and *EIF4EBP2*, but not *EIF4E*. Finally, we found that miR-483-5p reduced the level of phosphorylated eIF4E (pSer209eIF4E) but not total eIF4E. In conclusion, our study suggests that miR-483-5p's multi-targeting effect on the ERK1/ MKNK1 axis modulates the phosphorylation state of eIF4E. Unlike siRNA, miRNA can have multiple targets in the pathway, and thereby exploring the role of miR-483-5p in various cancer models may uncover therapeutic options.

## Introduction

Eukaryotic mRNA translation is an energy consuming process, and it occurs in three stages: initiation, elongation and termination. Among the well-understood mechanisms for mRNA translation initiation are: cap-dependent process (usually occurring under physiological conditions) and cap-independent process (usually occurring under stress/infection conditions), mediated by the internal ribosome entry site (IRES)^1–3^. In cap-dependent translation, eukaryotic translation initiation factor 4E (eIF4E) binds to the 7-methylguanosine 5′-triphosphate (m^7^GTP) at the 5′ end of mRNA^4^. eIF4E is a part of the multi-protein eIF4F complex that also comprises other proteins such as the scaffold protein eIF4G, and the RNA helicase eIF4A. eIF4F complex further recruits accessory 43S pre-initiation complex (containing 40S small ribosomal subunit, initiation factors such as eIF1, eIF1A, eIF3, eIF5, and the eIF2/Met-tRNAi/GTP ternary complex) that facilitates the detection of AUG start codon in the 5′UTR. Once 60S large ribosomal subunit binds to 43S pre-initiation complex, 80S ribosome is formed to initiate the peptide synthesis^2,5^.

**Figure 1:**
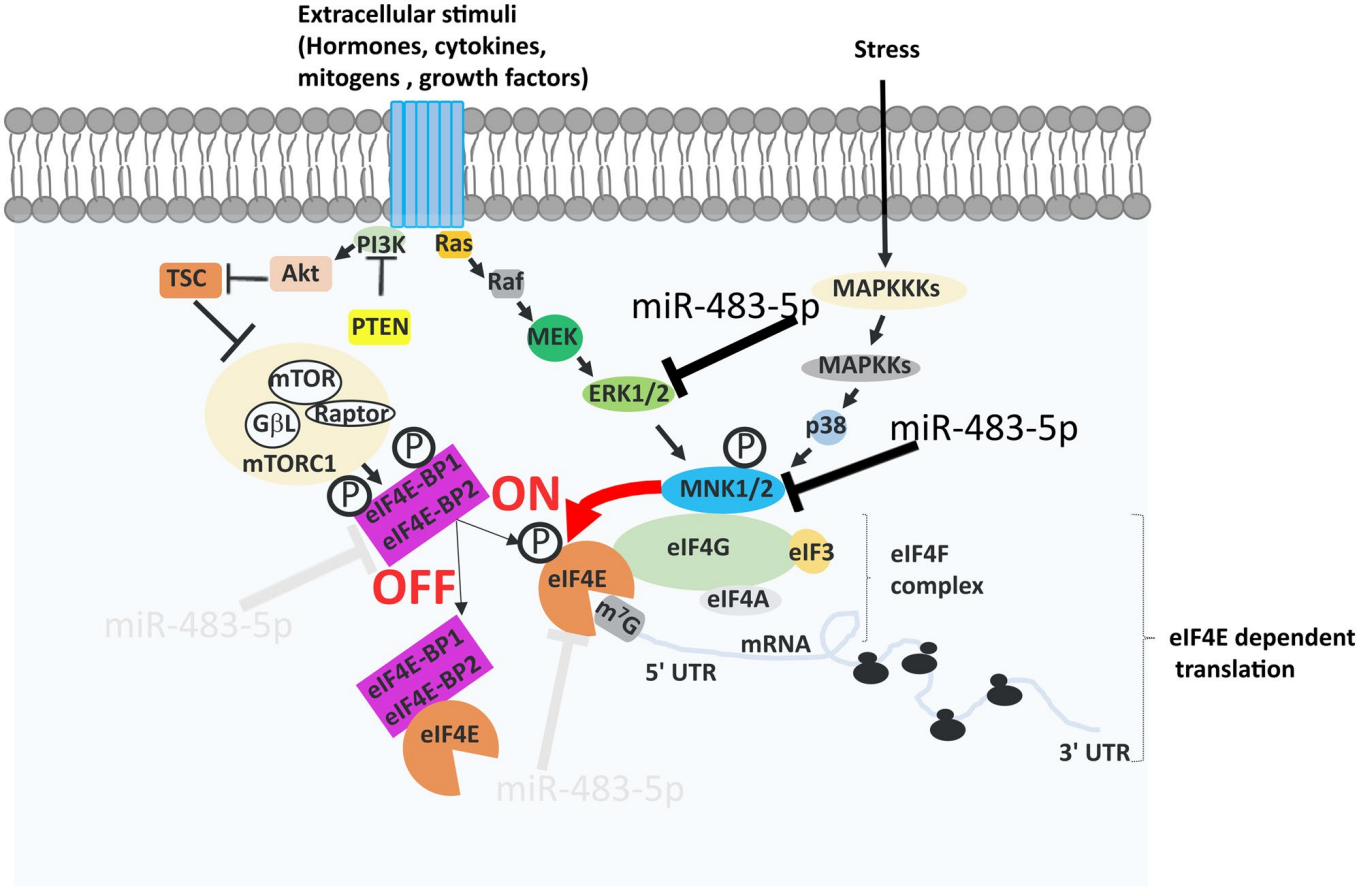
Signalling pathways in cap-dependent mRNA translation. Activation of Ras/MAPK pathway kinases (in response to mitogenic and stress signals) such as ERK and p38 leads to MKNK1/2 activation, and further phosphorylation of eIF4E at Ser209. Activation of PI3K pathway kinases (in response to extracellular stimuli) such as mTORC1 complexes, leads to the phosphorylation of the translation repressors eIF4E-BP1/2, thereby allowing eIF4E to form the eIF4F complex. Net cumulative effect of these two pathways determines the on / off switching of the cap dependent translation. Inhibitory regulation by miR-483-5p in cap-dependent mRNA translation pathway is shown (black—experimentally verified targets, light gray—predictions yet to be verified).

In cap-dependent translation the binding of eIF4E to the m^7^GTP is a crucial step limiting the rate of mRNA translation. Therefore, to reduce the aberrant translation, eIF4E is regulated by several mechanisms. First, at the transcription level by the MYC transcription factor^6,7^. Second, at the level of eIF4F complex formation, through eIF4E binding proteins (4E-BPs) that compete with eIF4G for binding to eIF4E thus limiting the eIF4F complex formation and in consequence decelerating the cap-dependent translation^8^. Alternatively, active mTORC1 signalling phosphorylates 4E-BP and causes its dissociation from eIF4E, thus allowing the formation of eIF4F complex and subsequently accelerating translation (Fig. 1)^8^. Third, eIF4E activity is regulated by phosphorylation at the serine 209^9^. Phosphorylation of eIF4E at the serine 209 leads to the increased global protein translation and is observed in various cancer types. It is known to regulate several pathways, including proliferation, apoptosis and metastasis^9–14^. MKNK (MNK) is the upstream kinase that phosphorylates eIF4E, thus targeting the MKNK-eIF4E axis reduce the level of eIF4E phosphorylation^15–20^. Recently, a review compiled various MKNK inhibitors studied to limit the eIF4E phosphorylation^15^. Also, several independent studies showed that even inhibition of the upstream kinase ERK1/2 regulates the ERK-MKNK-eIF4E axis and results in the reduction of eIF4E phosphorylation^21–23^.

microRNAs (miRNAs) are small noncoding RNAs (19–24 nucleotides) that regulate gene expression by post-transcriptional mechanisms and have been shown to reduce the overall protein synthesis by targeting the cap-dependent translation^24,25^. miRNAs are differentially expressed in several pathological conditions^26,27^. Previous studies by us and others have shown that miR-483-5p mediates ERK1 targeting^28,29^ and down-regulates the ERK1 phosphorylation in HEK293 cells. In a separate study, it was found that miR-483-5p targeted MKNK1 in Wilms’ tumour cells^30^. Because eIF4E phosphorylation was not examined in these studies, we wanted to explore the potential of miRNA to regulate eIF4E phosphorylation. We hypothesised that multi-targeting binding of miR-483-5p would synergistically affect the phosphorylation of both ERK1 and MKNK1, and lead to the repression of eIF4E phosphorylation (Fig. 1). In addition, in the current study, we explored the potential of miR-483-5p for regulation of 4E-BPs.

## Results

### Prediction and experimental investigation of miR-483-5p targets: *EIF4E, EIF4EBP1 *and *EIF4EBP2*

**Figure 2:**
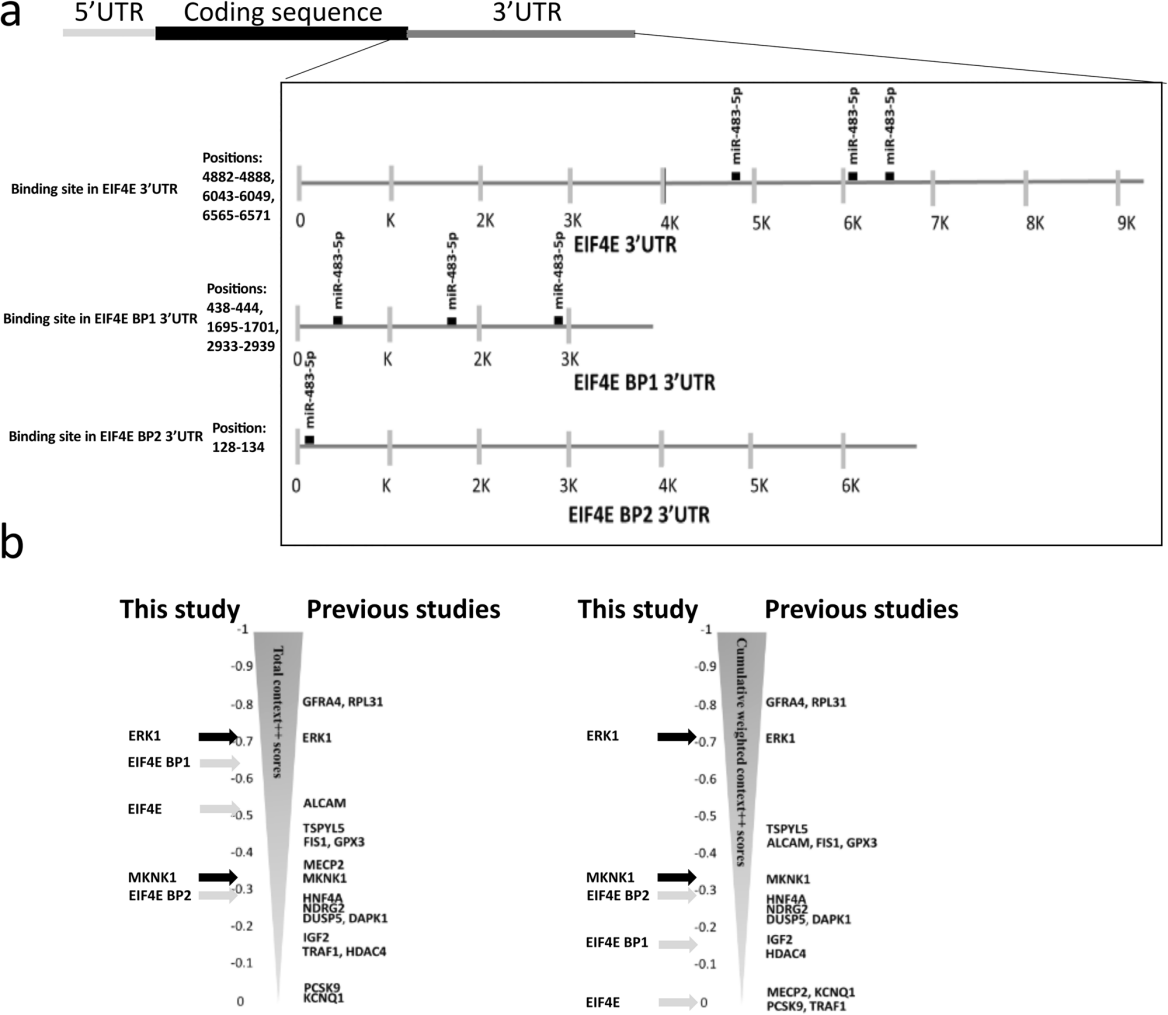
Prediction of miR-483-5p targets in cap-dependent mRNA translation pathway. (**a**) miR-483-5p predicted to bind 3′UTR of eIF4E mRNAs (3 sites), of EIFE 4BP1 mRNAs (3 sites) and EIF4E BP2 mRNAs (1 site). (**b**) Relative positions of targets out of 1818 targets based on predicted computational scores (total context++ score and cumulative weighted context++ score) from TargetScan database. Black arrows and gray arrows represent verified and novel targets investigated in this study, respectively. Other experimentally verified targets were positioned accordingly.

By searching the TargetScan database with an algorithm that finds binding sites for miRNA in the 3′ UTR of the mRNA, we found 1818 target mRNAs with varying strength of predicted binding to miR-483-5p. Among them 28 targets have been experimentally verified according to the Web of Science database by May 2023 [*GFRA4, ERK1, RPL31, ALCAM, TSPYL5, FIS1, GPX3, MeCP2, MKNK1, HNF4A, NDRG2, DUSP5, DAPK1, IGF2, TRAF1, HDAC4, PCSK9, KCNQ1, SRF, SOCS3, RAI16, TBL1X, RhoGDI1, CKB, NOTCH3, TGFB1, RBM5*, and *PIAS1*] that are known to be repressed by miR-483-5p (Fig. 2b). Interestingly, the experimental targets had varying total context++ scores, with the highest score (− 0.81) for *GFRA4* and *RPL31* and the lowest score (− 0.05) for *KCNQ1*, indicating the necessity for the experimental evaluation of predicted targets.

Within the listed 28 targets, present are those involved in the regulation of the translation process, kinases ERK1 and MKNK1/2. Affecting these kinases could impair the downstream targets of miR-483-5p, including *EIF4E*. Therefore, we decided to further investigate genes encoding eIF4E and eIF4E-binding proteins (4E-BP1, 4E-BP2) as targets of miR-483-5p. In the 3′UTR of *EIF4E* we found three binding sites for miR-483-5p at positions 4882–4888, 6043–6049, 6565–6571. These are poorly conserved 7mer-m8 type sites (exact match to positions 2–8 of the mature miRNA) with the cumulative weighted context++ score 0 and total context++ score − 0.53 (Fig. 2a,b). In the 3′UTR of *EIF4EBP1* we found three binding sites for miR-483-5p at positions 438–444, 1695–1701, 2933–2939. These are poorly conserved 7mer-m8 type sites (exact match to positions 2–8 of the mature miRNA) with the cumulative weighted context++ score − 0.16 and total context++ score − 0.65 (Fig. 2a,b). In the 3′UTR of *EIF4EBP2* we found one binding site for miR-483-5p at the position 128–134. This is poorly conserved 7mer-m8 type site (exact match to positions 2–8 of the mature miRNA) with the cumulative weighted context++ score − 0.29 and total context++ score − 0.29 (Fig. 2a, b).

**Figure 3:**
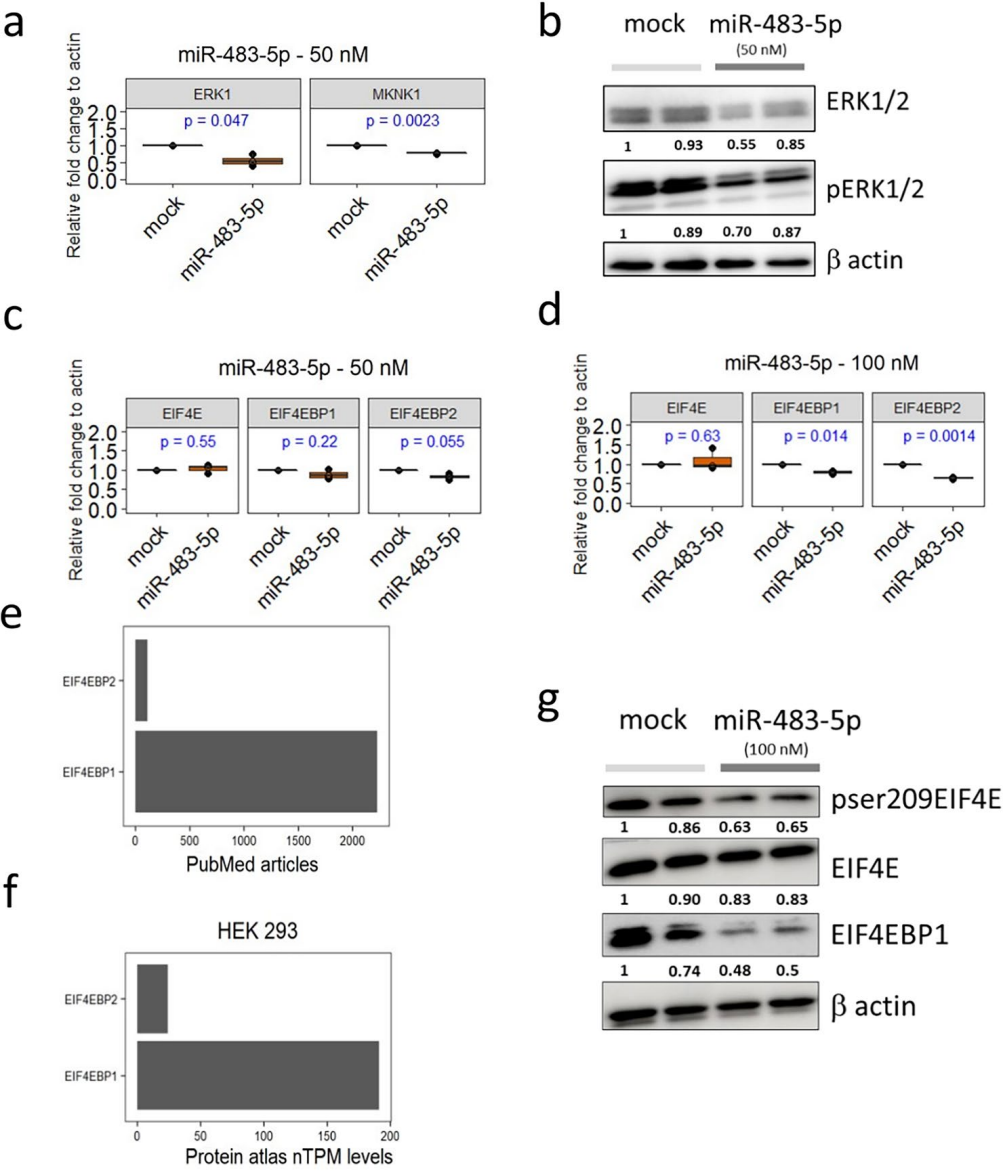
Reduction of pSer209eIF4E, 4E-BP1 by miR-483-5p. (**a**) RT-qPCR analysis of previously validated targets *ERK1* and *MKNK1* mRNA levels upon transfection with 50 nM of miR-483-5p mimic. (**b**) Representative immunoblots for pERK1/2 and ERK1/2 protein levels upon transfection with mock and miR-483-5p mimic at 50 nM. (**c**,**d**) RT-qPCR analysis of novel targets *EIF4E*, *EIF4EBP1* and *EIF4EBP2* mRNA levels upon transfection with 50 nM (**c**) and 100 nM (**d**) of miR-483-5p mimic. (**e**) Number of PubMed articles retrieved by May 2023 on 4E-BP1 and 4E-BP2. (**f**) Relative expression of 4E-BP1 and 4E-BP2 in HEK293 based on protein atlas database (proteinatlas.org). (**g**) Representative immunoblots for pSer209eIF4E, eIF4E and 4E-BP1 protein levels upon transfection with mock and miR-483-5p mimic at 100 nM. β-Actin (*ACTB*) was used as a reference for normalization in a-d and g. Uncropped blots are provided in supplementary material.

At first, we wanted to recapitulate the repression for the previously verified targets (*ERK1* and *MKNK1*) in HEK293. As expected, we observed that 50 nM miR-483-5p mimic significantly reduced ERK1 and MKNK1 mRNA levels in comparison to mock-transfected cells (Fig. 3a). Further we noticed decreased ERK1/2 and pERK1/2 protein levels in these conditions (Fig. 3b). We then asked whether miR-483-5p at 50 nM could repress hitherto not experimentally verified targets *EIF4E*, *EIF4EBP1* and *EIF4EBP2.* We noticed no change of the transcript level for *EIF4E*, slight but non-significant reduction for *EIF4EBP1* and significant reduction of *EIF4EBP2* (Fig. 3c)*.* Increasing the miR-483-5p mimic to 100 nM, resulted in no change in transcript levels for EIF4E, but significantly reduced transcript levels for *EIF4EBP1* and *EIF4EBP2* (Fig. 3d). Overall, our data indicate that transcript of both *EIF4EBP1* and *EIF4EBP2* is suppressed by miR-483-5p, but not that of *EIF4E*.

### miR-483-5p mediated reduction of pSer209eIF4E but not eIF4E levels

ERK1/2 and MKNK1 are kinases that phosphorylate their downstream targets including eIF4E. Therefore, we decided to investigate the level of phosphorylated protein eIF4E after transfection of HEK293 cells with miR-483-5p. For this purpose, we used antibody against total eIF4E and another antibody against phosphorylated eIF4E, specific to serine 209 (pSer209eIF4E). Indeed, immunoblotting experiments showed that at concentration of 100 nM miR-483-5p mimic reduced pSer209eIF4E protein level in comparison to mock transfected cells (Fig. 3g). On the other hand, transfection with miR-483-5p mimic had no effect on the levels of eIF4E (Fig. 3g). This observation is consistent with RT-qPCR analysis that showed unchanged levels of *EIF4E* mRNA (Fig. 3c,d) after miR-483-5p mimic transfection, while *ERK1* and *MKNK1* mRNA levels significantly decreased (Fig. 3a). Collectively, our results indicate that miR-483-5p indirectly reduces the level of phosphorylated eIF4E by downregulating the expression of ERK1/2 and MKNK1 kinases.

### miR-483-5p mediates reduction of 4E-BP1 protein

The other miR-483-5p targets we have selected were mRNAs encoding 4E-BP1 and 4E-BP2, two proteins involved in the regulation of cap-dependent translation process. 4E-BP1 is a well-studied protein that binds to eIF4E and thus inhibits the formation of eIF4F, which in consequence affects cap-dependent translation. PubMed search revealed 20 times more articles on 4E-BP1 compared to those on 4E-BP2 as of May 15th 2023 (Fig. 3e). Moreover, Protein atlas database indicated that the relative expression of 4E-BP1 is eightfold higher than 4E-BP2 in HEK293 cells (Fig. 3f). Due to low expression levels in HEK293 (Fig. 3f), limited literature showing insufficient characterization in translational regulation, and a lack of well-annotated antibodies against 4E-BP2 (https://www.citeab.com/antibodies/search?q=4ebp1,https://www.citeab.com/antibodies/search?q=4e+bp2 ), we did not explore protein levels of 4E-BP2 in this study, only mRNA levels (Fig. 3c,d). However, we evaluated 4E-BP1 protein levels in HEK293 cell lysates, using well-validated antibody against 4E-BP1. We found that miR-483-5p mimic at 100 nM reduced mRNA levels of both 4E-BP1 and 4E-BP2 (Fig. 3c,d) and lowered 4E-BP1 protein level in comparison to mock transfected cells (Fig. 3g). Overall, upon miR-483-5p transfection we found reduction of protein levels of pSer209eIF4E and of 4E-BP1.

### Multi targeting miR-483-5p vs single targeting siERK1: Different consequences on *C-MYC* and *CCND1* expression

**Figure 4:**
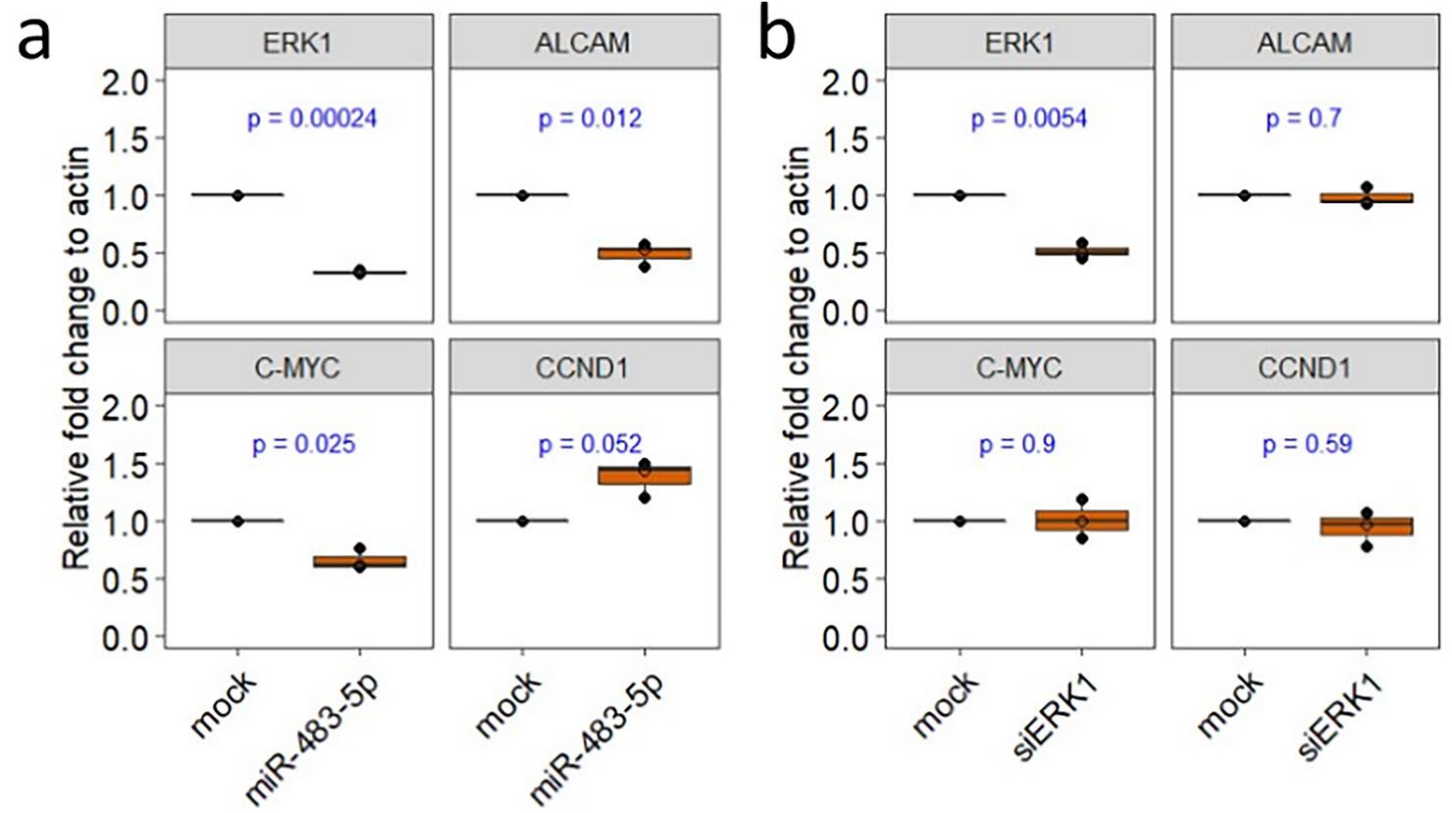
Multi-targeting potential of miR-483-5p compared to mono-targeting by siERK1. RT-qPCR analysis of *ERK1*, *ALCAM*, *C-MYC*, and *CCND1* mRNA levels upon transfection with 100 nM of miR-483-5p mimic (**a**) and 50 nM of siERK1 (**b**). β-Actin (*ACTB*) was used as a reference for normalization in RT-qPCR experiments.

Next, we evaluated the cellular consequences of miR-483-5p mediated down regulation of ERK1 and resulting reduced level of pSer209eIF4E. In these experiments we used *ERK1* and another experimentally validated target of miR-483-5p, Activated Leukocyte Cell Adhesion Molecule, *ALCAM* as positive reference controls to show the multi targeting potential of miR-483-5p (Fig. 2b). A recent study demonstrated that S209A substitution in eIF4E decreased the level of phosphorylated eIF4E and in consequence reduced the levels of both *C-MYC* mRNA and C-MYC protein^14^. Also in our case, after transfection of HEK293 cells with miR-483-5p, the level of *C-MYC* mRNA was reduced, which may be due to the decreased levels of pSer209eIF4E (Fig. 4a). We previously reported miR-483-5p mediated reduction of pERK1 in HEK293^28^, however its nuclear and cytoplasmic distribution is unknown. Interestingly, a study showed miR-483-5p mediated regulation of nucleus resident DUSP5^31^. Moreover, it is documented that DUSP5 loss increases nuclear ERK1/2 phosphorylation and expression of its nuclear targets^32^. Therefore, further we evaluated one such nuclear ERK target, cyclin D1 (CCND1), whose expression is enhanced by nuclear ERK activity^33,34^. We asked if miR-483-5p transfection would increase CCND1 expression. We noticed that it increases the *CCND1* mRNA levels upon miR-483-5p transfection (Fig. 4a). To further confirm that such observation is specific to the multi-targeting effect of miR-483-5p, we used siRNA specific to ERK1 (siERK1) as a control to evaluate the levels of *C-MYC* and *CCND1*. As expected, in siERK1 experiments, only *ERK1* mRNA levels were reduced but not *ALCAM, C-MYC,* and *CCND1* (Fig. 4b). Taken together, in HEK293 cells upon miR-483-5p transfection, multi targeting regulation by miR483-5p results in the reduced mRNA levels of *C-MYC* and enhanced mRNA levels of *CCND1*.

## Discussion

miRNAs are known to target multiple mRNAs through complementary base pairing^26,27^. This miRNA-mRNA interaction is complex and not all miRNA mediated mRNA targeting impacts the cellular phenotype. Some of the interactions counterbalance each other (mRNA targets considered weak) whereas other interactions (mRNA targets considered strong) consistently determine the cellular phenotype^35,36^. Furthermore, this complexity is enhanced by intrinsic feedback loops^37^ and dose specific response^38^. Therefore, there is still a need for extensive characterization of miRNA and its effect on predicted mRNA targets in different cell types.

Here we showed reduction in pSer209eIF4E and 4E-BP1 levels upon transfection with miR-483-5p mimic in HEK293 cells. The rationale for selecting miR-483-5p was based on our previous research, which highlighted its capability to target ERK1^28^. This finding aligns with other studies, establishing a consensus on miR-483-5p's regulation of ERK1^29,39^. Through the exploration of possible targets, we identified the robust potential of miR-483-5p to bind MKNK1 (Fig. 2b), a discovery supported by both our investigations (Fig. 3a) and those of others^30^. However, the impact of miR-483-5p on the downstream relays of the ERK1/MKNK1 axis, due to a cumulative effect on these two kinases, remains unclear. The ERK1/MKNK1 axis plays a crucial role in regulating the eIF4E signalling pathway, determining global protein levels. Under normal physiological conditions, the ERK pathway is activated by factors such as hormones, growth factors, and differentiation factors^40^. In pathological conditions, such as those associated with tumor-promoting factors, the ERK pathway is also activated. While the individual effects of the cascading activation signals are extensively studied for each kinase^41,42^, the well-established function of the ERK1/MKNK1 axis is to phosphorylate the mRNA cap-binding protein eIF4E^15,22,43,44^.

**Figure 5:**
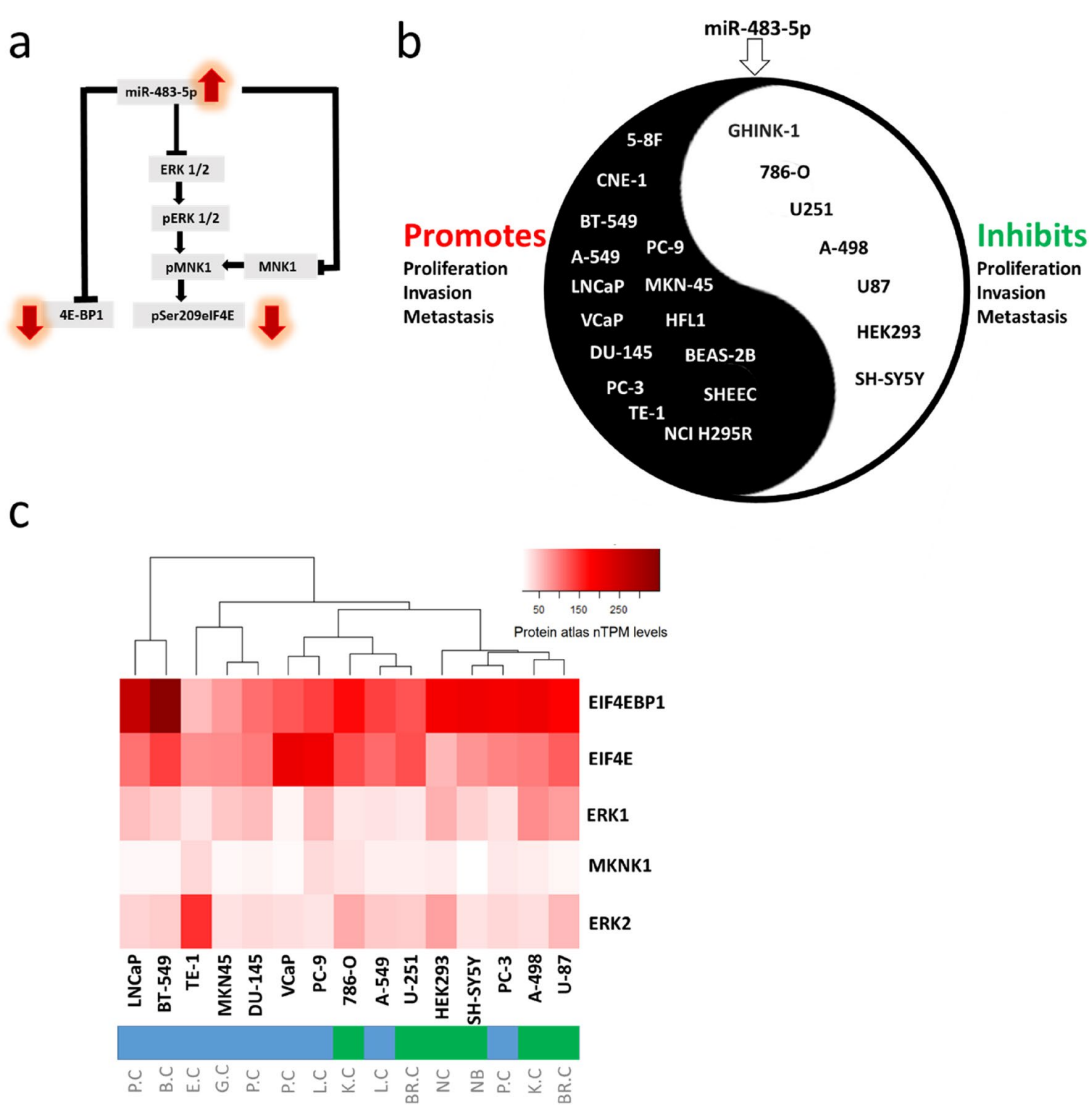
Cell to cell variability upon miR-483-5p transfection. (**a**) The scheme shows the pathway for the reduction of pSer209eIF4E and 4E-BP1 in HEK293. (**b**) Evidence from the literature for phenotypic variability among cell lines (promotion/inhibition of proliferation, invasion and metastasis) upon miR-483-5p transfection. (**c**) Protein atlas data showing variance in the relative protein levels targeted by miR-483-5p among different cell lines. *P.C* Prostate cancer, *B.C* Breast cancer, *E.C* Esophageal cancer, *G.C* Gastric cancer, *L.C* Lung cancer, *K.C* Kidney cancer, *NC* Non-cancerous, *NB* Neuroblastoma, *BR.C* Brain cancer.

In physiological contexts, miR-483-5p plays a role in preserving the function and identity of pancreatic β-cells, promoting adipogenesis in adipose tissue, and alleviating hyperlipidemia-associated fatty liver disease^45^. Dysregulation of miR-483-5p is associated with various diseases, including cancer^46,47^, type 2 diabetes, fatty liver disease, diabetic nephropathy, and neurological injury^45^. Recognizing that miR-483-5p regulates the ERK1/MKNK1 axis could be a crucial information for modulating both physiological and pathological conditions. This is the first study to report how miR-483-5p affects cellular levels of key proteins involved in global protein translation. The proposed mechanism of these effects are schematically shown in Fig. 5a. Notably, miR-483-5p is a microRNA dysregulated in various cancers, and the levels of both pSer209eIF4E and 4E-BP1 are also increased several-fold in different cancers^15,48^. Therefore this is of particular interest to further investigate the role of miR-483-5p in the regulation of pSer209eIF4E and 4E-BP1 in various cells. Such studies are important because they can aid to explain the basic mechanism of miRNA action and define the differences in cancer cells, allowing for the identification of novel therapeutic targets.

It is currently unknown if the observed miR-483-5p-driven repression of the levels of these two proteins is part of a buffering noise or a significant repression that determines the cellular phenotype. In order to determine the exact role of this mechanism in proliferation, invasion and metastasis, different cell lines need to be investigated especially that divergent phenotypes are observed upon miR-483-5p transfection of various cancer cell lines, as shown in Fig. 5b. In certain cell lines miR-483-5p promoted^46,49^ whereas in other cell lines it inhibited the proliferation, invasion and metastasis^29,47,50^. One possible mechanism for such divergent phenotypes could be due to the net outcome of the collective miRNA-mRNA interactions, probably stemming from the differences in available mRNA targets and their competition for miR-483-5p binding (Fig. 5b). Intriguingly, the protein atlas data is in line with literature evidence showing two different clusters (shown in green and blue) among the cell lines based on the protein signatures of miR-483-5p targets, at least those involved in the cap-dependent translation process (Fig. 5c). Nevertheless, such data should be validated both in vitro and in vivo across various cancer models to further examine phenotypic variability upon miR-483-5p overexpression.

The intriguing aspect of the results obtained in this study lies in the fact that miR-483-5p exerts control over two distinct routes: (1) the ERK1/MKNK1 axis and (2) EIF4EBP1. These routes, in essence, may counteract each other, leading to a delicate balance of pEIF4E levels. This balance, in turn, may play a pivotal role in determining the fate of translation processes. The intricate regulation of these pathways by miR-483-5p underscores the complexity and nuanced control exerted by this microRNA on protein translation. Additionally, miR-483-5p has multiple potential target mRNAs, and its multi-targeting effect can extend beyond the ERK1/MKNK1 axis. However, a limited number of miR-483-5p targets, as illustrated in Fig. 2, have undergone experimental verification. Notably, these validated targets are recognized players in cancer-related processes. The intricate network of pathways involving these targets suggests a high likelihood of interplay among them, potentially contributing to a discernible phenotype across diverse cancer types.

Previous studies showed that miR-455-3p, miR-15a and miR-141 mediated direct repression of eIF4E and suggested the tumour suppressing role of miRNAs in prostate cancer, renal cell carcinoma, and non-small cell lung cancer, respectively^24,51,52^. However, the previous studies have not addressed the issue of pSer209eIF4E levels. Collectively, in our study we show for the first time that miR-483-5p mediates reduction of pSer209eIF4E without affecting total eIF4E levels via direct regulation of the ERK1 and MKNK1.

At mRNA levels we noticed a reduction of *C-MYC* and an increase of *CCND1* after miR-483-5p transfections, but not after siERK1 transfections. These results emphasize the multi targeting potential of a miRNA, not achieved with siRNA. More importantly, it is yet to be investigated in different cells whether reduced eIF4E phosphorylation could have compensating effect in affecting the cap dependent translation of *CCND1*. Furthermore, to obtain deeper mechanistic insights, the cross talk between 4E-BP1-C-MYC^53^ and 4E-BP1-CCND1^54^ needs to be deciphered in individual cell types.

In our study, we used the standard HEK293 cell line, which is a model of an unaltered cell. This was to understand the physiological basis of miR-483-5p's influence on the phosphorylation of the cap-binding protein eIF4E by targeting the ERK1/MKNK1 axis. Our results provide a basis for the characterization of differences in the molecular processes responsible for pathological alterations. It is essential to expand on this finding, particularly in to the context of cancer, where the phosphorylation of the cap-binding protein eIF4E is notably high^11^ and dysregulation of miR-483-5p has been previously reported^55^. Taken together our results indicate that miR-483-5p mediated reduction of pSer209eIF4E and 4E-BP1 in HEK293. However, the underlying mechanisms differ significantly. The decrease in pSer209eIF4E levels can be attributed to the diminished kinases in the eIF4E phosphorylation pathway, a consequence of miR-483-5p binding to the mRNA of these kinases (ERK1 and MKNK1). In contrast, the reduction in 4E-BP1 levels is a direct result of miR-483-5p binding to 4E-BP1 mRNA. Moreover, we suggest the importance to evaluate pSer209eIF4E and 4E-BP1 levels upon miR-483-5p transfections among different cancer cell lines to understand the mechanisms behind the cellular phenotype, such as proliferation, invasion and metastasis. Furthermore, deciphering co-regulated targets of miR-483-5p, in conjunction with phosphorylated Ser209 eIF4E and 4E-BP1, is needed for unbiased understanding. Additionally, the assessment of off-target effects of miR-483-5p and the exploration of delivery modes for miR-483-5p in vivo are crucial steps towards comprehending its therapeutic applications. Before making conclusive statements about the potential therapeutic implications of miR-483-5p, it is essential to systematically classify the cell types or cancer models where miR-483-5p exerts inhibitory or promotional effects on proliferation and metastasis. Given that this observation is based on existing literature, it is equally important to reproduce the findings using different dosage titrations to gain comprehensive insights into the effects on the relevant targets and pathways. It's noteworthy that the dosage of miRNA can lead to varying effects, as evidenced by existing research^38^.Another important investigation is analysis of the patient prognosis associated with miR-483-5p levels. However, it is noteworthy that several studies have already been conducted to assess the prognostic significance of miR-483-5p across various cancer types^56–61^. The continuation of this research can provide an insight into prognostic biomarker potential of miR-483-5p.

## Methods

### Cell culture

Human embryonic kidney 293 (HEK293) obtained from the American Type Culture Collection (ATCC) were grown in Eagle’s Minimum Essential Medium (EMEM) (Sigma-Aldrich) supplemented with 10% foetal bovine serum (FBS) (Sigma-Aldrich), 2 mM L-glutamine and 100 mg/mL penicillin/streptomycin. Cells were cultured at 37 °C in a humidified atmosphere of 5% CO_2_.

### miRNA and siRNA transfection

miR-483-5p mirVana miRNA mimic (#4464066) was used (Ambion). Mock transfections were performed with only Opti-MEM medium and Lipofectamine 2000 without mimics. In miRNA transfection experiments, mock was used as baseline for evaluating the effect of miR-483-5p mimic. In siRNA transfection experiments, siERK1 (#AM16708) (Ambion) was used to silence ERK1. In siRNA transfection experiments, mock was used as baseline for evaluating the effect of siERK1. In all these experiments, cells were seeded at 1 × 10^6^ cells/well in 12-well plates 24 h prior to transfection. Cells were grown to 60–80% confluence and transfected with miRNA mimics (up to 100 nM) or siERK1 (50 nM). Cells were harvested at 48 h post-transfection for the quantitative real-time polymerase chain reaction (RT-qPCR) and immunoblotting.

### RNA isolation and RT-qPCR

Total RNA was isolated from cells using the Total RNA Mini isolation kit (A&A Biotechnology) according to the manufacturer’s instructions. RNA was quantified spectrophotometrically and its quality was analysed using A_260_/A_280_ ratio (DeNovix). Up to 150 ng of RNA was used to obtain cDNA using the High-Capacity cDNA Reverse Transcription Kit (Thermo Fisher Scientific). Quantitative PCR was performed on LightCycler 480 II System (Roche). Briefly, 2 μl of cDNA (obtained from 150 ng of RNA), 4 μl of each mRNA specific primers (5 pmol of forward and reverse) (Supplementary material), 10 μl of Maxima SYBR Green qPCR Master Mix (2X) (Thermo Fisher Scientific) were mixed in a 20 μl reaction and run with a thermal profile of an initial 10 min melting step at 95 °C, followed by 45 cycles at 95 °C for 10 s, 60 °C for 10 s and 72 °C for 10 s. The relative fold change of mRNAs was normalized to β-actin mRNA by 2^−ΔΔCt^ method^62^.

### Immunoblotting

HEK293 lysates were obtained using RIPA lysis buffer (Sigma Aldrich) with phosphatase and protease inhibitor cocktails (Roche). The protein concentrations in the samples were determined using the Pierce BCA protein assay kit (Thermo Fisher Scientific). A total of 20 µg of protein samples diluted in sample buffer containing 2-mercaptoethanol (Sigma-Aldrich), denatured for 10 min at 95 °C, were loaded in each well and separated by sodium dodecyl sulfate–polyacrylamide gel electrophoresis (SDS-PAGE) in a 10 or15% polyacrylamide gel cast using the TGX FastCast acrylamide kit (Bio-Rad). After electrophoretic separations, proteins were transferred to polyvinylidene fluoride (PVDF) membrane using a transfer unit (Bio-Rad) at 80 V for 1 h. After transfer, the PVDF membranes were blocked with 5% bovine serum albumin (BSA) for 2 h and incubated overnight at 4 °C with primary antibodies. These include: anti-pSer209eIF4E (Cell Signaling Technology #9741), anti- eIF4E (Cell Signaling Technology, #9742, anti-4E-BP1(Cell Signaling Technology #9452) and anti-β-actin (Cell Signaling Technology #8H10D10). Detection of the immunoreaction was performed with a Clarity western enhanced chemiluminescence substrate kit (Bio-Rad) using ChemiDoc MP imaging system (Bio-Rad).

### Software and statistical analysis

To retrieve predicted targets for miR-483-5p, we used TargetScan v7.2^63^. To retrieve relative protein expression levels, we used protein atlas (proteinatlas.org). Immunoblot densitometry was performed using Fiji software^64^. All statistical analyses were performed with R software v3.6.2 (cran.r-project.org). For RT-qPCR experiments unpaired student t test was used. Raw p value was mentioned, and the data are shown as mean ± standard error.

### Supplementary Information

Supplementary Information.

## Data Availability

All data generated or analyzed during this study are included in this published article and its Supplementary information files.
