# Supplementary material for: miR-483-5p orchestrates the initiation of protein synthesis by facilitating the decrease in phosphorylated Ser209eIF4E and 4E-BP1 levels
Source: Sci Rep. 2024 Feb 20;14:4237. doi: 10.1038/s41598-024-54154-1 (PMC10879198; doi:10.1038/s41598-024-54154-1)

miR-483-5p orchestrates the initiation of protein synthesis by facilitating the decrease in phosphorylated Ser209eIF4E and 4E-BP1 levels

Siranjeevi Nagaraj[1,3], Anna Stankiewicz-Drogon[2], Edward Darzynkiewicz[1,2], Urszula Wojda[3*], Renata Grzela[1,2*]

[1]Interdisciplinary Laboratory of Molecular Biology and Biophysics, Centre of New Technologies, University of Warsaw, 02-097 Warsaw, Poland

[2]Division of Biophysics, Institute of Experimental Physics, Faculty of Physics, University of Warsaw, Pasteura 5, 02-093 Warsaw, Poland

[3]Laboratory of Preclinical Testing of Higher Standard, Nencki Institute of Experimental Biology of Polish Academy of Sciences, Pasteur 3, 02-093 Warsaw, Poland

* Correspondence: rgrzela@fuw.edu.pl; u.wojda@nencki.edu.pl

Supplementary table 1. Primers used in this study

Target	Primer	Sequence (5’ to 3’)
ERK1	Forward	CGCTACACGCAGTTGCAGTACA
ERK1	Reverse	AAGCGCAGCAGGATCTGGA
MKNK1	Forward	AGATGGGCAGTAGCGAACC
MKNK1	Reverse	AGCAATTCAGAGGTCAGCTTG
EIF4E	Forward	CGGAATCTAATCAGGAGGTTGC
EIF4E	Reverse	GATCAGCCGCAGGTTTGC
EIF4E BP1	Forward	GCAATAGCCCAGAAGATAAGCG
EIF4E BP1	Reverse	CCTTGGTAGTGCTCCACAC
EIF4E BP2	Forward	CCCAATATCCCAGGAGTCACT
EIF4E BP2	Reverse	CTTGCAGGAGAGTCAGATGTC
ALCAM	Forward	CAGATTGGTGATGCCCTAC
ALCAM	Reverse	GAGCAGTTTCGCAGACATAG
C-MYC	Forward	TGAGGAGACACCGCCCAC
C-MYC	Reverse	CAACATCGATTTCTTCCTCATCTTC
CCND1	Forward	GCTGCGAAGTGGAAACCATC
CCND1	Reverse	CCTCCTTCTGCACACATTTGAA
ACTB	Forward	GCCGAGGACTTTGATTGC
ACTB	Reverse	CTGTGTGGACTTGGGAGAG

Full original images of immunoblots pertaining the figure 3b is shown (lane 2-5 is highlighted in the red box)

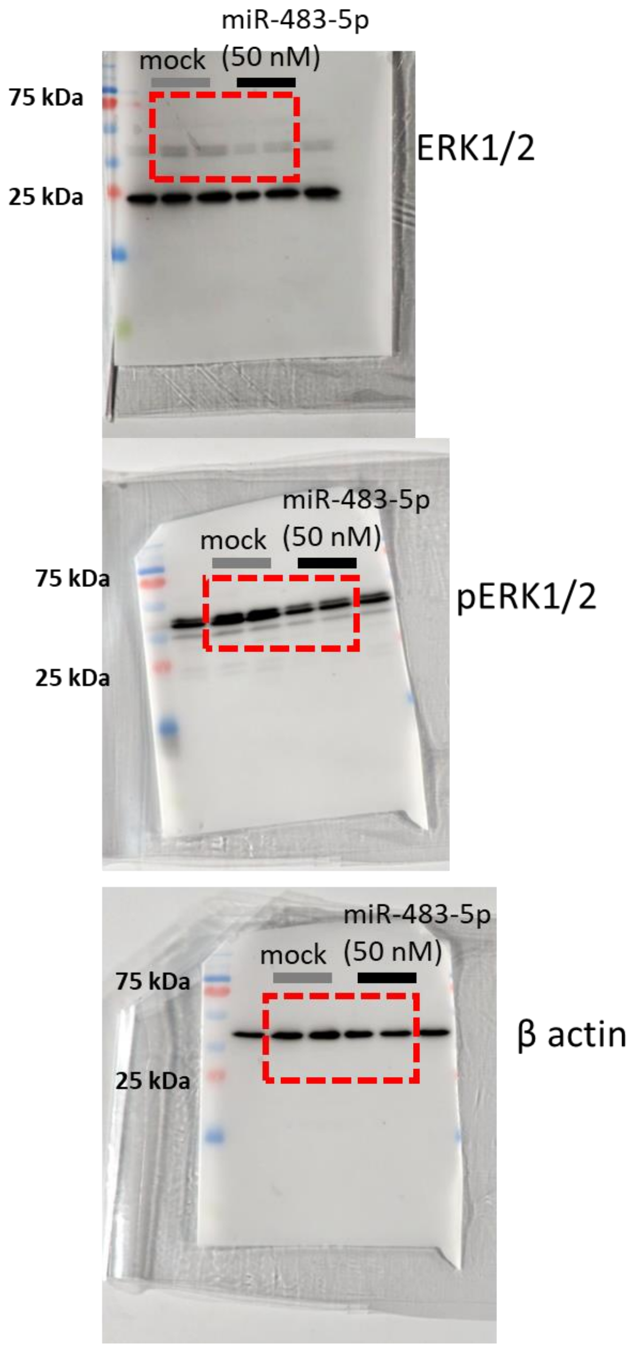

Full original images of immunoblots pertaining the figure 3g is shown (lane 2-5 is highlighted in the red box)

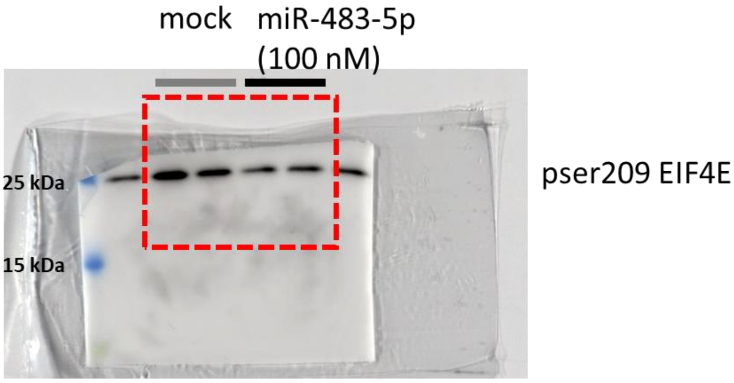

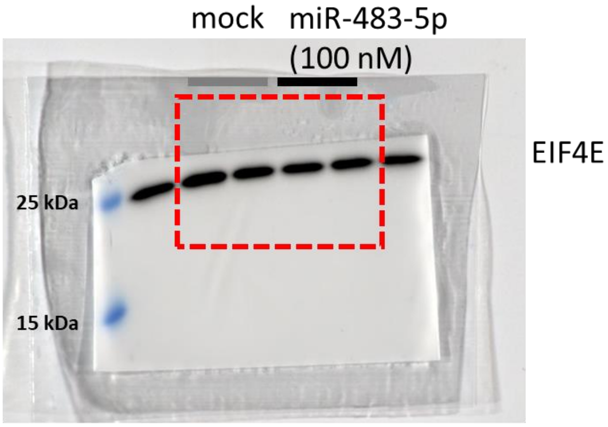

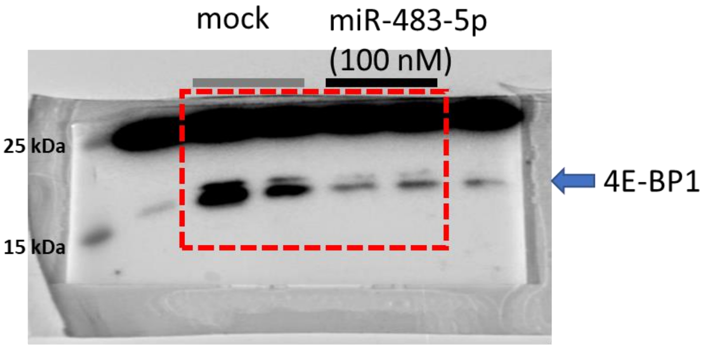

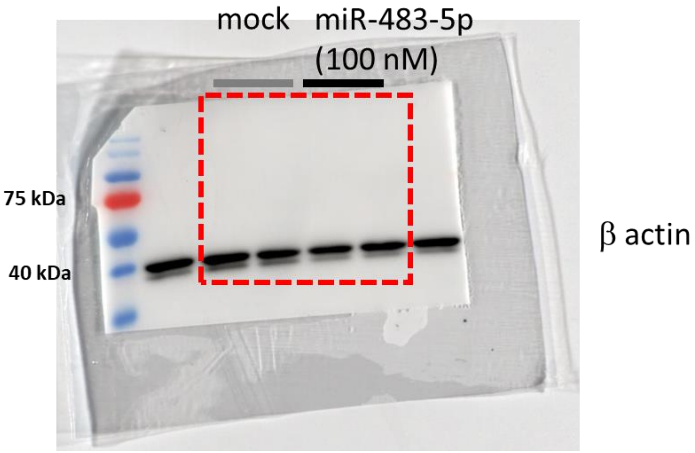

Supplement: Supplementary file 1 — Supplementary Information. [file 41598_2024_54154_MOESM1_ESM.pdf]
